# Immunological characteristics govern the transition of COVID-19 to endemicity

**DOI:** 10.1126/science.abe6522

**Published:** 2021-01-12

**Authors:** Jennie S. Lavine, Ottar N. Bjornstad, Rustom Antia

**Affiliations:** 1 Department of Biology, Emory University, Atlanta, GA 30322, USA.; 2 Department of Biology and Center for Infectious Disease Dynamics, The Pennsylvania State University, University Park, PA 16802, USA.

## Abstract

We are currently faced with the question of how the severity of infection with severe acute respiratory syndrome coronavirus 2 (SARS-CoV-2) may change in the years ahead. Our analysis of immunological and epidemiological data on endemic human coronaviruses (HCoVs) shows that infection-blocking immunity wanes rapidly but that disease-reducing immunity is long-lived. Our model, incorporating these components of immunity, recapitulates both the current severity of SARS-CoV-2 infection and the benign nature of HCoVs, suggesting that once the endemic phase is reached and primary exposure is in childhood, SARS-CoV-2 may be no more virulent than the common cold. We predict a different outcome for an emergent coronavirus that causes severe disease in children. These results reinforce the importance of behavioral containment during pandemic vaccine rollout, while prompting us to evaluate scenarios for continuing vaccination in the endemic phase.

## Taming a pandemic

One year after its emergence, severe acute respiratory syndrome coronavirus 2 (SARS-CoV-2) has become so widespread that there is little hope of elimination. There are, however, several other human coronaviruses that are endemic and cause multiple reinfections that engender sufficient immunity to protect against severe adult disease. By making assumptions about acquired immunity from its already endemic relatives, Lavine *et al.* developed a model with which to analyze the trajectory of SARS-CoV-2 into endemicity. The model accounts for SARS-CoV-2's age-structured disease profile and assesses the impact of vaccination. The transition from epidemic to endemic dynamics is associated with a shift in the age distribution of primary infections to younger age groups, which in turn depends on how fast the virus spreads. Longer-lasting sterilizing immunity will slow the transition to endemicity. Depending on the type of immune response it engenders, a vaccine could accelerate establishment of a state of mild disease endemicity.

*Science*, this issue p. 741

## Teaser

The transition from epidemic to endemic dynamics of SARS-CoV-2 is associated with a shift in the age distribution of primary infections to younger age groups.

Humans have regularly been threatened by emerging pathogens that kill a substantial fraction of all people born. Recent decades have seen multiple challenges from acute virus infections, including severe acute respiratory syndrome (SARS), Middle East respiratory syndrome (MERS), Hendra, Nipah, and Ebola. Fortunately, all were locally contained. When containment is not immediately successful, as is likely for the novel betacoronavirus severe acute respiratory syndrome coronavirus 2 (SARS-CoV-2) (*1*, *2*), we need to understand and plan for the transition to endemicity and continued circulation, with possible changes in disease severity owing to virus evolution and buildup of host immunity and resistance.

SARS-CoV-2 is an emerging virus that causes COVID-19. The virus has a high basic reproductive number (*R*_0_) and is transmissible during the asymptomatic phase of infection, both of which make it hard to control (*3*). However, there are six other coronaviruses with known human chains of transmission, which may provide clues to future scenarios for the current pandemic. There are four human coronaviruses (HCoVs) that circulate endemically around the globe; these cause only mild symptoms and are not a considerable public health burden (*4*). Two other HCoV strains, SARS-CoV-1 and MERS-CoV, emerged in recent decades and have higher case fatality ratios (CFRs) and higher infection fatality ratios (IFRs) than COVID-19 but were contained and thus never spread widely (*5*, *6*).

We propose a model to explore the potential changes in both transmission and disease severity of emerging HCoVs through the transition to endemicity. We focus on SARS-CoV-2 and discuss how the conclusions would differ for emerging coronaviruses more akin to SARS-CoV-1 and MERS-CoV. Our hypothesis is that all HCoVs elicit immunity with similar characteristics, and the current acute public health problem is a consequence of epidemic emergence into an immunologically naïve population in which older age groups with no previous exposure are most vulnerable to severe disease. We use our estimates of immunological and epidemiological parameters for endemic HCoVs to develop a quantitative model for endemic transmission of a virus with SARS-CoV-2–like characteristics, including the age dependence of severity. Our model explicitly considers three separate measures for immune efficacy that wane at different rates (fig. S1).

Building on ideas from the vaccine modeling literature, we suggest that immunity may provide protection in three ways (*7*). In its most robust form, sterilizing immunity can prevent a pathogen from replicating, thereby rendering the host refractory to reinfection. We term this property immune efficacy with respect to susceptibility (IE_S_). If immunity does not prevent reinfection, it may still attenuate pathology due to reinfection (IE_P_) and/or reduce transmissibility or infectiousness (IE_I_). Indeed, experimental reexposure studies on endemic HCoVs provide evidence that the three immune efficacies do not wane at the same rate (*8*, *9*). Callow *et al.*’s experimental study (*8*) shows that reinfection is possible within one year (relatively short IE_S_); however, upon reinfection, symptoms are mild (high IE_P_) and the virus is cleared more quickly (moderate IE_I_). Details on the derivation of the model can be found in section 2 of the supplementary materials (SM).

**Fig. 1:**
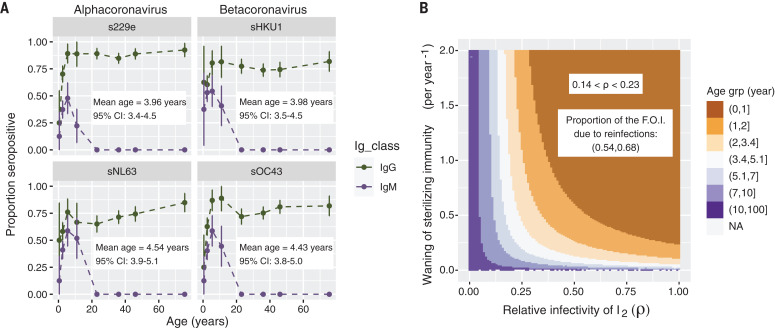
A low mean age of primary infection suggests that partially transmissible reinfections are common. (**A**) Mean proportion seropositive for IgG (green, top lines) and IgM (purple, bottom lines) against the four endemic HCoV strains [dots connected by dashed lines; vertical lines represent the 95% confidence interval (CI); data from (*10*)]. The mean age of primary infection (MAPI) based on IgM data with 95% CI is shown in the inset of each panel (see SM for details). (**B**) MAPI as a function of waning of sterilizing immunity ω (*y* axis) and transmissibility of reinfections ρ (*x* axis). The MAPI was calculated from the equilibrium dynamics of the model shown in fig. S1 and supplementary equations 3 to 9 with a plausible basic reproductive number (*R*_0_ = 5), 0 < ω < 2, and 0 < ρ < 1. See SM section 2.1 for details. The inset shows the plausible combinations of values of ρ and ω consistent with the MAPI for HCoVs estimated in (A). F.O.I., force of infection; I_2_, reinfection. [See fig. S1 for parallel figures calculated at extreme plausible values for *R*_0_ (i.e., *R*_0_ = 2 and 10).]

We reanalyze a detailed dataset that estimates age-specific seroprevalence on the basis of both immunoglobulin M (IgM; acute response) and IgG (long-term memory) against all four circulating HCoVs in children and adults (*10*) to estimate parameter ranges for transmission and waning of immunity (Fig. 1A). The rapid rise in both IgM and IgG seroprevalence indicates that primary infection with all four endemic HCoV strains happens early in life, and our analysis of these data gives us an estimate for the mean age of primary infection (MAPI) between 3.4 and 5.1 years, with almost everyone infected by age 15 (see SM section 1 for details). The absence of detectable IgM titers in any individual over the age of 15 years suggests that reinfection of adults causes a recall response, indicating that while HCoV-specific immunity may wane, it is not lost. Whether immunity would wane to naïve levels in the absence of high pathogen circulation remains an open question.

For most people to be infected so early in life—younger even than measles in the pre-vaccine era—the attack rate must exceed transmission from primary infections alone. The model shows that a high attack rate can arise from a combination of high transmissibility from primary infections (i.e., high *R*_0_), waning of sterilizing immunity, and substantial transmission from reinfections in older individuals. The rapid waning of sterilizing immunity is also reported in experimental HCoV infections of humans, which showed that reinfection is possible 1 year after an earlier infection, albeit with milder symptoms (IE_P_) and a shorter duration (IE_I_) (*8*). Figure 1B shows the plausible combinations of waning immunity and transmission from reinfected individuals that are required to produce the MAPI observed in Fig. 1A, based on steady-state infection levels (see SM section 2.1 for details). Table 1 shows the ranges of the parameters used in our simulations.

**Table 1:** Characteristics of coronavirus-immunity interactions and relevant parameter ranges.

**Characteristic and symbol**	**Estimates from literature**	**Value (range)**	**Citations**
Primary infectious period (1/γ)	≥5.6 days ~10 days	9 days	(*8*) (*30*)
Primary transmissibility [*R*_0_ = β/(γ + μ)]	4 to 9	2 to 10	(*31*)
Secondary transmissibility (relative to primary, ρ)	0.35 0.04 to 0.97	0 to 1	(*8*) Fig. 2 and fig. S2
Duration of sterilizing immunity (1/ω)	0.91 years 1.67 years 0.5 to 2 years	0.5 to 10 years	(*8*) and SM section 5 (*8*) and SM section 5 (*11*, *32*)
Relative pathology of reinfections	Mild	–	(*8*)
Age-specific IFR (primary infections)	SARS MERS COVID-19	See Fig. 3	(*33*) (*5*) (*34*)

**Fig. 2:**
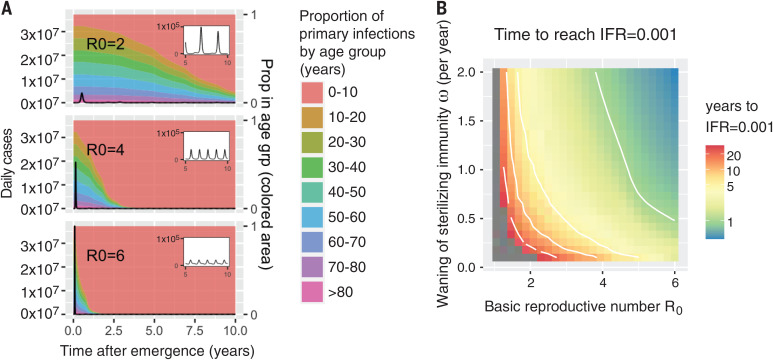
The time scale of the transition from epidemic to endemic dynamics for emerging coronaviruses depends on *R*_0_ and the rate of immune waning. Transition from epidemic to endemic dynamics for emerging HCoVs, simulated from an extension of the model presented in fig. S1 that includes age structure. Demographic characteristics (age distribution, birth, and age-specific death rates) are taken from the United States, and seasonality is incorporated via a sinusoidal forcing function (see SM section 2.2). Weak social distancing is approximated by *R*_0_ = 2. (See figs. S9 to S11 for strong social distancing results, *R*_0_ < 1.5.) (**A**) Daily number of new infections (black line; calculations in SM section 2.3). An initial peak is followed by a low-incidence endemic state (years 5 to 10 shown in the inset). A higher *R*_0_ results in a larger and faster initial epidemic and a more rapid transition to endemic dynamics. The proportion of primary cases in different age groups changes over time (plotted in different colors), and the transition from epidemic to endemic dynamics results in primary cases being restricted to younger age groups. Parameters for simulations: ω = 1 and ρ = 0.7. (**B**) Time for the average IFR (6-month moving average) to fall to 0.001, which is the IFR associated with seasonal influenza. Gray areas represent simulations where the IFR did not reach 0.001 within 30 years. The time to IFR = 0.001 decreases as the transmissibility (*R*_0_) increases and the duration of sterilizing immunity becomes shorter. Results are shown for ρ = 0.7. See SM section 2.3 and figs. S4 to S7 for sensitivity analyses and model specifications.

**Fig. 3:**
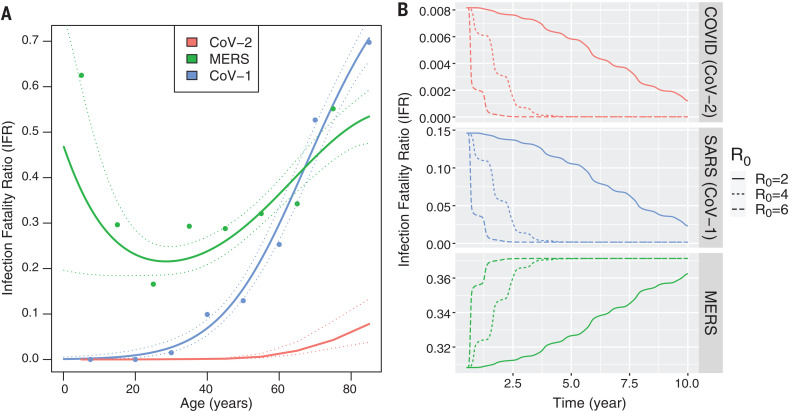
The overall infection fatality ratio (IFR) of emerging coronaviruses once they reach endemicity is strongly influenced by the IFR of young children in the initial epidemic. The age dependence of the IFR determines how the overall IFR changes during the transition from epidemic to endemic dynamics for emerging HCoVs. (**A**) Age dependence of the IFRs for the three emerging HCoVs. Primary infections with MERS-CoV and SARS-CoV-1 are consistently symptomatic, and the IFR and CFR are therefore assumed to be the same. SARS-CoV-1 and SARS-CoV-2 have J-shaped profiles, with a monotonic increase in IFR with age. The age-specific IFR for MERS-CoV is U-shaped, with high mortality in both the young and old age groups. Details of the statistical smoothing are described in SM section 6. (**B**) The overall IFR changes during the transition to endemic dynamics. These calculations assume that deaths due to reinfections are negligible. We relax this assumption to allow for a slower buildup of immunity and possible death due to secondary infection in figs. S5 to S9 and show that the qualitative results do not change.

At the beginning of an outbreak, the age distribution of cases mirrors that of the population (Fig. 2A). However, once the demographics of infection reaches a steady state, our model predicts that primary cases occur almost entirely in babies and young children, who, in the case of COVID-19, experience a low CFR and a concomitantly low IFR. Reinfections in older individuals are predicted to be common during the endemic phase and to contribute to transmission, but in this steady-state population, older individuals, who would be at risk for severe disease from a primary infection, have acquired disease-reducing immunity after infection during childhood. The top panel of Fig. 3B illustrates how the overall IFR for SARS-CoV-2 drops drastically, eventually falling below that of seasonal influenza (~0.001) once the endemic steady-state is reached.

The time it takes to complete the shift in IFR as endemicity develops depends on both transmission (*R*_0_) and loss of immunity [waning of sterilizing immunity (ω) and transmissibility of reinfections (ρ)], as shown in Fig. 2B and fig. S4. The transition from epidemic to endemic dynamics is associated with a shift in the age distribution of primary infections to lower age groups (Fig. 2A). This transition may take anywhere from a few years to a few decades, depending on how quickly the pathogen spreads. The rate of spread, measured by *R*_0_, is determined by a combination of viral properties and the frequency of social contacts and may therefore be reduced by social distancing. The top panel in Fig. 2A shows the effect of reducing *R*_0_ to 2, whereas the middle and bottom panels show the dynamics for higher *R*_0_, which are more akin to those of SARS-CoV-2 in the absence of control measures. If transmission is high, the model predicts a high case load and high death rate in earlier years following emergence (Fig. 2 and fig. S5). We see that, as might be expected, longer-lasting sterilizing immunity slows down the transition to endemicity (Fig. 2B). These results are robust to a more biologically realistic distribution for the duration of sterilizing immunity and the possibility that the generation of protective immunity requires more than one infection (see SM section 3 and figs. S5 to S9).

Slowing down the epidemic through social distancing measures that reduce *R*_0_ to close to 1 flattens the curve, thus delaying infections and preventing most deaths from happening early on, affording critical time for the development of an effective vaccine (fig. S10). If vaccine-induced IE_S_ and IE_P_ immunity is similar to that induced by HCoV infections, the vaccine may usher in the endemic regime more quickly. The model code (see the acknowledgments) provides a flexible scaffolding for studying alternative vaccination scenarios. Notably, the model predicts that once the endemic state is reached, mass vaccination may no longer be necessary to save lives (see SM section 4 and fig. S11).

We can extend our predictions to two other potentially emerging coronavirus infections, SARS and MERS. Our model predicts that in the endemic state, the IFR of a circulating HCoV depends primarily on the severity of childhood infections. In the case of SARS-CoV-1, which is more pathogenic than SARS-CoV-2, we still expect a low disease burden in the endemic phase, because SARS-CoV-1, like SARS-CoV-2, has a low IFR in young people (Fig. 3). However, data suggest that not all emerging HCoVs follow this optimistic pattern; the overall IFR of an endemic MERS-like virus would not decrease during the transition to endemicity, as seen in Fig. 3B, and this is because disease severity (and IFR) is high in children, the age group expected to experience the bulk of primary cases during the endemic phase. In the endemic phase, a vaccination program against MERS would therefore be necessary to avoid excess mortality (fig. S11).

The key result from our model framework that explicitly recognizes that functional immunity to reinfection, disease, and shedding are different is that, in contrast with infections that are severe in childhood, SARS-CoV-2 could join the ranks of mild, cold-causing endemic HCoVs in the long run. A critical prediction is that the severity of emergent HCoVs once they reach endemicity depends only on the severity of infection in children (Fig. 3), because all available evidence suggests that immunity to HCoVs has short IE_S_ and moderate IE_I_, leading to frequent reinfection throughout adulthood (*11*, *12*), but strong IE_P_ such that childhood infection provides protection from pathology upon reinfection in adulthood, as evidenced by the rarity of severe infections or detectable IgM titers in adults. Strain-specific virulence factors, such as the shared cellular receptor, angiotensin-converting enzyme 2 (ACE-2), to which SARS-CoV-1, SARS-CoV-2, and the endemic strain NL63 all bind (*13*–*16*), may affect the CFR during the emergence phase but have little impact on the severity of disease in the endemic phase. Because the four endemic HCoVs have been globally circulating for a long time and almost everyone is infected at a young age, we cannot ascertain how much pathology would result from a primary or even a secondary case of any of these in an elderly or otherwise vulnerable person.

The key insights come from how our model explicitly incorporates different components of immunological protection with respect to susceptibility, pathology, and infectivity (IE_S_, IE_P_, and IE_I_, respectively) and their different rates of waning. In our analysis, we hypothesized that these components of immunity for SARS-CoV-2 are comparable to those of endemic HCoVs, and this needs to be determined. Additionally, during the transition to endemicity, we need to consider how the immune efficacies depend on primary and secondary infections across ages (*17*) and how responses differ between vaccination and natural infection.

Longitudinal analysis of SARS patients provides an opportunity to measure the durability of immune memory in the absence of reexposure. The only long-term study we know of that follows SARS-CoV-1–specific antibodies suggests that they wane faster than antibodies to other live viruses and vaccines such as measles, mumps, rubella, and smallpox (*18*) and fall below the threshold of detection in 6 years (*19*). In contrast to antibody responses, memory T cells persist for much longer periods (*19*, *20*) and confer protection in animal model systems (*21*).

We further consider the effects of strain variation both for natural infection and vaccination. Strain variation and antibody escape may occur in endemic strains (*22*); however, the fact that symptoms are mild suggests that immunity induced by previously seen strains is nonetheless strong enough to prevent severe disease. Indeed, among HCoVs, frequent reinfections appear to boost immunity against related strains (*12*). However, the effect of strain variation may differ for vaccine-induced immunity, especially in light of the narrower epitope repertoire of many currently authorized vaccines.

If frequent boosting of immunity by ongoing virus circulation is required to maintain protection from pathology, then it may be best for the vaccine to mimic natural immunity insofar as preventing pathology without blocking ongoing virus circulation. Preliminary results suggest the adenovirus-based vaccine is better at preventing severe than mild or asymptomatic infections (*23*), and it will be important to collect similar data for the other vaccines. Should the vaccine cause a major reduction in transmission, it might be important to consider strategies that target delivery to older individuals for whom infection can cause higher morbidity and mortality, while allowing natural immunity and transmission to be maintained in younger individuals. During the transition to endemicity, primary SARS-CoV-2 infections will frequently occur in older individuals, and we need to determine whether immunity induced by infection or vaccination in adulthood is similar to that produced by natural infections in childhood. Thus far, there have been few reinfections reported with SARS-CoV-2, and disease severity has varied (*24*); the only population-level study of reinfection that we are aware of estimates a low rate of reinfection in the first 6 months after primary infection and mild disease upon reinfection (*25*), but further analysis and monitoring are vital.

The findings presented here suggest that using symptoms as a surveillance tool to curb the spread of SARS-CoV-2 will become more difficult, as milder reinfections increasingly contribute to chains of transmission and population-level attack rates. In addition, infection or vaccination may protect against disease but not provide the type of transmission-blocking immunity that allows for shielding (*26*) or the generation of long-term herd immunity (*2*).

The details of the change in overall IFR through the transient period will be affected by a wide array of factors, such as age-specific human contact rates (*27*) and susceptibility to infection (*28*) as well as improvement in treatment protocols, hospital capacity, and virus evolution. The qualitative result of mild disease in the endemic phase is robust to these complexities, but quantitative predictions for the transient phase will depend on a careful consideration of these realities and how they interact with the dynamics of infection and components of immunity (*29*).

The changes in the IFR over time predicted by the model have implications for vaccination strategy against current and future emerging HCoVs. Social distancing and an effective vaccine are critical for control during a virgin epidemic and the transition out of it, but once we enter the endemic phase, mass vaccination may no longer be necessary. The necessity for continual vaccination will depend on the age-dependence of the IFR. If primary infections of children are mild (as for SARS-CoV-1 and SARS-CoV-2), continued vaccination may not be needed as primary cases recede to mild childhood sniffles. If, on the other hand, primary infection in children is severe (as for MERS), then vaccination of children will need to be continued.

From an ecological and evolutionary perspective, our study opens the door to questions regarding the within-host and between-host dynamics of human immunity and pathogen populations in the face of immune efficacies with different kinetics. It also opens the question of how these immune efficacies interplay with strain cross-immunity, which is likely relevant within the alpha- and betacoronaviruses. Considering data and model predictions from emergence through endemicity of HCoVs revealed a framework for understanding immunity and vaccination that may apply to a variety of infections, such as respiratory syncytial virus and seasonal influenza, which share similar age distributions and immune responses.

## Supplementary Materials

science.sciencemag.org/content/371/6530/741/suppl/DC1 Supplementary Text Figs. S1 to S13 References (*36*–*42*) MDAR Reproducibility Checklist

## Appendix

View/request a protocol for this paper from *Bio-protocol*.
